# A use case of ChatGPT: summary of an expert panel discussion on electronic health records and implementation science

**DOI:** 10.3389/fdgth.2024.1426057

**Published:** 2024-10-24

**Authors:** Seppo T. Rinne, Julian Brunner, Timothy P. Hogan, Jacqueline M. Ferguson, Drew A. Helmer, Sylvia J. Hysong, Grace McKee, Amanda Midboe, Megan E. Shepherd-Banigan, A. Rani Elwy

**Affiliations:** ^1^ Center for Healthcare Organization & Implementation Research, VA Bedford Healthcare System, Bedford, MA, United States; ^2^ Pulmonary and Critical Care Medicine, Dartmouth-Hitchcock Medical Center, Lebanon, NH, United States; ^3^ Center for the Study of Healthcare Innovation, Implementation, and Policy, VA Greater Los Angeles Healthcare System, Los Angeles, CA, United States; ^4^ Center for Innovation to Implementation, Veterans Affairs Palo Alto Health Care System, Menlo Park, CA, United States; ^5^ Center for Innovations in Quality, Effectiveness and Safety, Michael E. DeBakey VA Medical Center, Houston, TX, United States; ^6^ Department of Medicine, Baylor College of Medicine, Houston, TX, United States; ^7^ Measurement Science QUERI, San Francisco VA Medical Center, San Francisco, CA, United States; ^8^ Department of Medicine, University of California San Francisco, San Francisco, VA, United States; ^9^ VA HSR&D Center for Innovation to Implementation (Ci2i), VA Palo Alto Health Care System, Menlo Park, CA, United States; ^10^ Department of Public Health Sciences, School of Medicine, University of California, Davis, CA, United States; ^11^ Department of Population Health Sciences, Durham VA Health Care System, Durham, NC, United States; ^12^ Department of Population Health Sciences, Duke University, Durham, NC, United States

**Keywords:** artificial intelligence, large language models, expert panel discussion, electronic health records, implementation science

## Abstract

**Objective:**

Artificial intelligence (AI) is revolutionizing healthcare, but less is known about how it may facilitate methodological innovations in research settings. In this manuscript, we describe a novel use of AI in summarizing and reporting qualitative data generated from an expert panel discussion about the role of electronic health records (EHRs) in implementation science.

**Materials and methods:**

15 implementation scientists participated in an hour-long expert panel discussion addressing how EHRs can support implementation strategies, measure implementation outcomes, and influence implementation science. Notes from the discussion were synthesized by ChatGPT (a large language model—LLM) to generate a manuscript summarizing the discussion, which was later revised by participants. We also surveyed participants on their experience with the process.

**Results:**

Panelists identified implementation strategies and outcome measures that can be readily supported by EHRs and noted that implementation science will need to evolve to assess future EHR advancements. The ChatGPT-generated summary of the panel discussion was generally regarded as an efficient means to offer a high-level overview of the discussion, although participants felt it lacked nuance and context. Extensive editing was required to contextualize the LLM-generated text and situate it in relevant literature.

**Discussion and conclusions:**

Our qualitative findings highlight the central role EHRs can play in supporting implementation science, which may require additional informatics and implementation expertise and a different way to think about the combined fields. Our experience using ChatGPT as a research methods innovation was mixed and underscores the need for close supervision and attentive human involvement.

## Introduction

With rapid technology advancement, the landscape of clinical care and research is transforming to incorporate artificial intelligence (AI) as a tool to improve patient outcomes and advance research methods. Yet, there is limited empirical data on how to best harness technological innovations and avoid unintended consequences in healthcare. Implementation research is crucial to systematically understand, assess, and support these transformative changes. We sought to examine AI as a research tool for summarizing and reporting qualitative data from an expert panel discussion on electronic health records (EHRs) and implementation science to determine the feasibility and efficiency of AI for future research studies.

Large language models (LLMs), such as ChatGPT, use AI to generate natural language text, and they are garnering increasing attention for their potential applications in research (1, 2). Because LLMs can quickly and fluently summarize text, they hold promise for synthesizing qualitative data from group discussions. Several websites, blogs, and instructional videos describe using LLMs to summarize team meetings (3–5), but existing research has not explored the quality of these summaries or how they could help synthesize expert panel discussions.

In this manuscript, we describe the process of using ChatGPT as an AI-driven research tool to summarize and synthesize an expert panel discussion on EHRs and implementation science(a field that is focused on increasing uptake and use of evidence-based practices in real-world settings). Specifically, we sought to examine how current EHRs and future EHR innovations could support implementation strategies and implementation outcomes. Implementation strategies are the methods or techniques used to enhance the adoption, implementation, and sustainability of clinical practices (6, 7). Implementation outcomes are defined as the effects of deliberate and purposive actions to implement new treatments, practices, and services, such as acceptability, costs, feasibility, fidelity, adoption, and sustainability (8).

EHRs have been widely adopted across US healthcare systems in response to the Health Information Technology for Economic and Clinical Health (HITECH) Act of 2009 (9). This technology has transformed healthcare delivery by providing real-time access to comprehensive patient information, optimizing workflows, facilitating communication among healthcare teams, and driving medical practice modernization (10). These capabilities present key opportunities for supporting implementation research as the field evolves and grows, although there have not been comprehensive efforts to highlight the potential for EHRs to contribute to implementation science.

By focusing on both the content of an expert panel discussion on EHR and implementation science and the process of using LLMs to summarize and report on the discussion, this manuscript seeks to shed light on two important and timely technologies impacting implementation science. Throughout the manuscript, we use subheadings “EHRs and implementation science” and “Application of an LLM to support expert panels” to separately describe the methods, results, and discussion related to each focus. When appropriate, we highlight synergies between these two topics.

## Methods

### EHRs and implementation science

This 1-hour, in person expert panel session was part of a larger Veterans Affairs (VA) Quality Enhancement Research Initiative (QUERI) meeting dedicated to implementation science topics. Fifteen VA health services researchers and implementation science experts participated in the session, which aimed to discuss the role of the EHR in implementation science research. Participants included several clinicians (two physicians, a pharmacist, a nurse, and a clinical psychologist) as well as numerous PhD level research scientists. The session incorporated Proctor's Implementation Outcomes framework (8) and Expert Recommendations for Implementing Change (ERIC) implementation strategies categorized by Waltz et al. (11) to address the following questions: (1) “How can the EHR support implementation strategies?”; (2) “How can the EHR assess implementation outcomes?”; and (3) “How will future EHRs further support implementation science?”

Participants separated into three breakout groups to discuss each of these questions and report back to all session participants. For each group, a dedicated participant scribe summarized and recorded the responses to the questions. As the discussion progressed, scribes emailed the responses to the session lead (STR), who then added them to a pre-written template (Supplementary File S1) that was used as input for ChatGPT to generate a full scientific manuscript. The template specifically stated, “Do not include references” to avoid well-documented concerns about LLMs producing erroneous citations (12). To keep discussions focused on the topics of EHRs and implementation research, participants were not made aware that their responses would be fed into ChatGPT until after the manuscript was generated at the close of the expert panel discussion. All participants agreed to using this data for publication. The study was part of a quality improvement initiative that was designated as non-research by the VA Boston Healthcare System Institutional Review Board.

### Application of an LLM as a research tool

The specific language model used was ChatGPT-3.5 architecture version May 25, 2023 (13). The generated manuscript (Supplementary File S2) was distributed to the participants via email, along with an anonymous online survey to collect feedback on the session and perceptions of using ChatGPT for summarization and reporting. The survey included Likert-type scale questions addressing the quality of the ChatGPT-generated synthesis, whether it captured key discussion points, and their comfort with future use of ChatGPT in similar situations. These were accompanied by three open-ended questions: (1) How would you describe your overall experience with the use of ChatGPT in this session?; (2) What did you find most beneficial about the use of ChatGPT in this session?; and (3) Was there anything about the use of ChatGPT in the session that you found challenging or problematic? Quantitative data was analyzed using descriptive statistics. We reviewed these responses and developed descriptive summaries (14), including brief quotations to illustrate key perspectives from survey participants.

The manuscript was revised with MS Word “Track Changes” to include survey results, add citations, update methods, and correct errors. We then distributed the manuscript to all session participants and asked them to revise it as they would with other manuscript drafts. To further distinguish ChatGPT from human-generated text, we asked all participants to not use LLMs to write their revisions. Supplementary File S3 is the revised manuscript with all Track Changes from session participants. The session lead (STR) finalized the manuscript based on the Track Changes recommendations, included a discussion on the revision process, and circulated to session participants for final approval.

## Results

### Conference session: EHRs and implementation science

#### EHRs and implementation strategies

Participants described EHRs as a powerful tool that could complement and facilitate most implementation strategies. Data from the EHR can engage participants in the implementation process and inform implementation efforts. Audit and feedback of EHR data was a frequently cited example of how EHRs can produce dashboards and data visualization that can engage clinicians in intervention efforts. Another example related to tailoring interventions to context based on patient, provider, and clinic characteristics that are readily available in the EHR. The EHR can also be used to train, educate, and support users. Participants cited examples that would use EHR communication channels to target key patient populations (via patient portals) and specific providers (via alerts, clinical notes, and focused messages). Participants went on to discuss using the EHR as a tool to impact clinical practice and provider behavior. The integration of decision support systems within the EHR was seen as particularly valuable, as it can facilitate real-time access to clinical guidelines and prompts, supporting clinicians in delivering guideline-concordant care. Finally, the EHR can impact strategies that extend beyond the technology itself. EHRs can redesign clinical workflows, impact clinical roles, and influence surrounding infrastructure. Participants highlighted the EHR's ability to streamline and optimize clinical processes, allowing for more efficient and effective implementation of evidence-based interventions.

#### EHRs and implementation outcomes

Participants noted that structured EHR data could be used to assess some implementation outcomes, including adoption, acceptability, fidelity, penetration (i.e., reach), and sustainability, if relevant data is captured by the EHR. For example, mental health care that requires multiple visits can be tracked by clinical encounters. Implementation cost may also be more easily assessed because most EHRs have been designed to capture clinical billing. Meta data on EHR use (e.g., time spent on documentation, use of specific EHR functions) is automatically captured in most EHR systems, and may offer information on specific implementation outcomes (e.g., if an EHR-based intervention is used by providers). Visual trends of structured data can be easily accessed, and presenting these outcome data can be particularly effective in driving practice and policy.

Unstructured data is more difficult to collect and analyze. Although EHRs capture a wealth of information, much of it is in free-text format, requiring manual processing and analysis. Participants highlighted the need for developing methods to effectively extract and analyze unstructured data, which would enhance the EHR's potential for supporting implementation science research and could help assess additional implementation outcomes (e.g., intervention acceptability could be reported in documentation about relevant clinical encounters). Expert panelists opined that existing applications of natural language processing tools and LLMs are often not advanced enough to effectively capture these outcomes, and when appropriate it may be necessary to change the EHR to include structured data on implementation outcomes. These changes can drive clinical practice, although they could have unintended consequences. For example, pain scores were integrated into most EHRs as a vital sign (alongside heart rate, blood pressure, and respiratory rate) to assess patient satisfaction with pain management, although this change contributed to increased opioid use in response to the data (15) and may have had contributed to the opioid crisis.

#### Future EHRs and implementation science

Participants acknowledged that existing EHRs have not fully lived up to their potential in facilitating efficient, high-quality, and patient-centered care. Future systems must help realize these goals. EHR changes should prioritize improved usability and user experience while also incorporating patient priorities in care planning. Participants highlighted the potential for generative artificial intelligence (AI) and LLMs to facilitate these changes through automated clinical documentation, clinical data synthesis and interpretation, improved decision support, and more advanced population health management. These tools have already begun to support direct patient interactions (e.g., automated secure message responses) (16), and future systems must identify appropriate use of LLMs in patient care. Implementation science will play a critical role in contributing to and evaluating these changes, including the need for ethical considerations and the involvement of human expertise in applications of LLMs and generative AI in clinical care.

Increasingly, data will be drawn from diverse sources, including wearables and remote monitoring. These changes can help implementation researchers access and use relevant EHR data for implementation research, and the field of implementation science may evolve to focus more on data management and analysis than on people management (i.e., implementing evidence-based interventions may involve more direct interactions with AI than trying to change provider behavior). Combined expertise in the fields of clinical informatics and implementation science is needed to support and assess future EHR changes.

### Application of an LLM as a research tool

#### Survey of participant perspectives

Survey responses were received from 12 of the 15 session participants (80%). 11 (92%) rated the quality of the synthesis produced by ChatGPT as positive or very positive. 11 (92%) agreed that “The ChatGPT-generated summary captured the key points from the discussion,” and 11 (92%) reported that they would be somewhat or very comfortable with a similar use of ChatGPT in future expert panel and consensus panel sessions.

All survey respondents wrote free text responses to open ended questions. These responses were generally positive, emphasizing the speed with which the summary was generated, “allowing better feedback from participants while meeting was fresh in their minds” and noting the surprising degree of coherence and clarity of the summary. Concerns included reliance on a single notetaker to capture nuances of the discussion, the need for transparency in distinguishing human vs. AI-generated content, and insufficient “authenticity and originality,” with one participant elaborating that it, may not capture the nuanced discourse that took place during the meeting but is “probably as good as a contractor,” an alternative approach that is often used during these types of meetings.

### Manuscript development

The ChatGPT generated summary of our session discussion followed the format of a scientific publication (Supplementary File S2), although we identified several challenges with the presentation that required extensive editing prior to submission (Supplementary File S3 presents the manuscript with tracked changes). First, the focus of the manuscript conflated the content of the session discussion (EHRs and implementation science) with the process of using ChatGPT as a research tool to summarize and report on the expert panel discussion, resulting in a manuscript that lacked a clear narrative. We believe both these components are important, and we made major revisions to clearly delineate the two elements and highlight synergies when appropriate. A second major challenge with the draft was that the results section was very brief, lacked organization, and did not adequately describe the important information included in the scribe-generated prompts. We expanded these results considerably, organizing them based on major concepts discussed during the session, and included additional information that was discussed during the session but not entered as ChatGPT prompts. A third challenge was that some of the generated text was not grounded in the content of the session and may reflect a tendency for LLMs to draw not only on from entered prompts, but also from other sources to generate text (12). We revised the manuscript to ensure stated results were a reflection of the session discussion. Finally, the manuscript discussion did not include context on prior literature. As noted in the methods, the ChatGPT prompt specifically stated “do not include references,” and we needed to make major revisions to contextualize the findings and add associated references.

## Discussion

We present an exemplary and novel case of how LLMs and generative AI can be used as a research tool to summarize and report on qualitative data generated from an expert panel discussion focusing on the role of EHRs in implementation science. We present both the content of this qualitative summary and the process of using ChatGPT for this purpose. The EHR and implementation science discussion yielded important insights, including specific examples of how EHRs can support implementation strategies, measure implementation outcomes, and influence the future of implementation science. The ChatGPT generated summary of this discussion provided an efficient research instrument for capturing the overview of the session's insights, and participants generally had positive impressions of applying LLMs for this purpose, although expert panelists had concerns that the summary did not adequately represent subtleties of the session discussion. We needed to make extensive changes to the manuscript to ground the text in the session discussion and draw out critical insights that could inform the application of this technology for implementation research.

### EHRs and implementation science

The expert panel session on EHRs and implementation science highlighted a central role for EHRs to support diverse implementation strategies and assess structured implementation outcome data. Our discussion aligns with a broad literature base that has repeatedly used EHRs in implementation science. Prior research substantiates our discussion that EHR could augment nearly all ERIC implementation strategies (7), including by using EHR data to engage providers (17, 18), facilitating communication (19–21), and introducing new technology-based tools (e.g., clinical decision support systems) that support implementation (22–24). There is also extensive implementation research that relies on structured EHR data to assess relevant outcomes (25–27), although prior literature corroborates the panel discussion that it is challenging to assess implementation outcomes, especially when relying on unstructured data (28).

The close link between EHRs and implementation science reflects the degree to which EHRs are not just a technology, but also increasingly play a fundamental role in care delivery. As EHRs continue to evolve, implementation science will need to evolve to assess these changes and support future care delivery. AI represents a disruptive technology that will transform care delivery with increasing automation of clinical documentation (29–31), clinical decisions (32–35), and direct patient interactions (36, 37). These changes underscore the need for additional expertise in informatics and implementation science to draw on the best evidence from these fields. Existing conceptual frameworks may need to be adapted to reflect the synergies between EHRs and implementation science, and new frameworks will likely be needed as EHRs and implementation science evolve.

### Application of an LLM as a research tool

There are clear advantages of using LLMs like ChatGPT as a research instrument for summarizing qualitative data generated from an expert panel discussion. Generative AI tools can provide a rapid digestion and presentation of the session discussion, which allows for a quick dissemination and review with session participants while the discussion is still fresh in their minds. Participants generally had a positive view of this LLM application, and many indicated that the summary adequately captured high-level findings, although there were concerns that it did not effectively account form more subtle details about the tone and content of the discussion. We noted major challenges with the ChatGPT-generated manuscript that required comprehensive editing to authentically present the session discussion. ChatGPT did not capture nuanced meanings or accurately represent the intent behind certain statements, and it failed to extract underlying concepts or themes to produce meaningful findings that could inform future practice, policy, and research. More concerningly, we observed instances in which ChatGPT generated text that was not based on the entered prompts and seemed to reflect other sources of information. We acknowledge that some of these challenges could be the result of variability in scribe-generated summaries, which may have been overcome by different methods of collecting expert panel discussion data and more detailed and directed prompts. Even with more attention to data collection and prompt generation, we submit that LLMs are inadequate to summarize expert panel discussion without close oversight and attention to authenticity.

Although we had hoped to generate a rapid manuscript that relied heavily on ChatGPT with minimal author effort, the reality was that revising the generated text was time consuming and required painstaking attention to detail to ensure that the final product accurately presented the session discussion and offered novel insights. We still believe that there may be a place for LLMs to support expert panel discussions, but we would refine our approach in several ways. First, we would include expert participants in planning LLM use, including the development and refinement of the template prompt. In our session, participants were not aware that their responses would be fed into ChatGPT, which was intended to maintain the discussion focus on EHRs and implementation science and present a surprise use case of LLMs as a research tool, but this approach did not prepare participants or engage them in designing appropriate LLM use. Second, we would consider additional methods of capturing the content of expert panel discussions, including with more structured templates or incorporate automated transcription and analysis tools. Third, we would use LLMs only to summarize the session discussion rather than generating a full manuscript. LLM use should be focused and supervised (2). Maintaining a focus on summarizing the session discussion would allow participants to scrutinize the summary and could push respondents to clarify their perspectives, thereby drawing out new inferences. Fourth, we would ensure sessions have sufficient time to review LLM-generated summaries with other panelists as a member checking approach to explore credibility of the results (38). Authors may still draft a manuscript on the process after the session, although we found that LLM use was inadequate for producing a high quality manuscript, and we rewrote virtually all the LLM-generated text.

It is also important to recognize that this study represents a relatively simple use case with straightforward data. The discussion centered around specific questions related to the role of EHRs in supporting implementation strategies and assessing implementation outcomes, and we collected breakout group notes directly from participants. In more complex scenarios that involve free flowing conversations (e.g., via automated transcription) or discussions involving conflicting perspectives, LLMs will likely encounter additional challenges, although as these technologies advance, they may be more adept at summarizing this complex data.

We must highlight several limitations with both the expert panel discussion and the application of LLMs as a qualitative research tool. First, the expert panel session involved a relatively small number of participants, and their responses do not represent the breadth of perspectives within the field of informatics and implementation science. The session's duration was limited to one hour, which may have constrained the depth of discussion on certain topics. Furthermore, the reliance on breakout group summary notes may have overlooked valuable insights that were expressed during small group discussions. Regarding LLM use, we acknowledge that the generated text is influenced by the ChatGPT 3.5 version used. LLMs are in a state of rapid development, and future versions may produce different results, which could influence their role in supporting expert panel discussions. The generated text is also influenced by the entered prompts, and more detailed and directed prompts would have produced a different manuscript.

## Conclusion

In conclusion, this manuscript presents a use case of ChatGPT as an example of an AI-based qualitative research tool to summarize and report on an expert panel discussion on EHRs and implementation science. This discussion yielded important insights on the central role EHRs can play in supporting implementation science, and we describe how future EHR changes may impact implementation science. Our discussion noted the need for additional informatics and implementation expertise and a different way to think about the combined fields. Our experience using ChatGPT to summarize and report on this discussion also yielded important information. The generated text offered a rapid and efficient means of presenting the session discussion, although the results lacked depth, nuance, and context. We found that the generated manuscript required extensive edits to situate it in existing literature and emphasize insights that could inform practice, policy, and research. Taken together, these findings underscore the critical role that implementation science must play to assess and refine technology use in clinical care and research.

## Data Availability

The raw data supporting the conclusions of this article will be made available by the authors, without undue reservation.

## References

[1] Cascella M Montomoli J Bellini V Bignami E. Evaluating the feasibility of ChatGPT in healthcare: an analysis of multiple clinical and research scenarios. J Med Syst. (2023) 47(1):33. 10.1007/s10916-023-01925-4 36869927 PMC9985086

[2] Sallam M. ChatGPT utility in healthcare education, research, and practice: systematic review on the promising perspectives and valid concerns. Healthcare (Basel). (2023) 11(6):887. 10.3390/healthcare11060887 36981544 PMC10048148

[3] McCue TJ. ChatGPT Hack for Summarizing Your Work. Forbes (2023). Available online at: https://www.forbes.com/sites/tjmccue/2023/01/26/chatgpt-hack-for-summarizing-your-work (accessed August 28, 2023).

[4] Pionk J. Use ChatGPT for Mundane Tasks Like Summarizing Meeting Notes and Formatting. LinkedIn (2023). Available online at: https://www.linkedin.com/pulse/use-chatgpt-mundane-tasks-like-summarizing-meeting-jerome-pionk/ (accessed August 28, 2023)

[5] Proper Project Management. Meeting Summaries in Minutes with ChatGPT. YouTube (2023). Available online at: https://www.youtube.com/watch?v=_GA08fyN9aQ (accessed August 28, 2023)

[6] Proctor EK Powell BJ McMillen JC. Implementation strategies: recommendations for specifying and reporting. Implement Sci. (2013) 8:139. 10.1186/1748-5908-8-139 24289295 PMC3882890

[7] Powell BJ Waltz TJ Chinman MJ Damschroder LJ Smith JL Matthieau MM A refined compilation of implementation strategies: results from the expert recommendations for implementing change (ERIC) project. Implement Sci. (2015) 10:21. 10.1186/s13012-015-0209-1 25889199 PMC4328074

[8] Proctor E Silmere H Raghavan R Hovmand P Aarons G Bunger A Outcomes for implementation research: conceptual distinctions, measurement challenges, and research agenda. Adm Policy Ment Health. (2011) 38(2):65–76. 10.1007/s10488-010-0319-7 20957426 PMC3068522

[9] Adler-Milstein J Jha AK. HITECH act drove large gains in hospital electronic health record adoption. Health Aff (Millwood). (2017) 36(8):1416–22. 10.1377/hlthaff.2016.1651 28784734

[10] Uslu A Stausberg J. Value of the electronic medical record for hospital care: update from the literature. J Med Internet Res. (2021) 23(12):e26323. 10.2196/26323 34941544 PMC8738989

[11] Waltz TJ Powell BJ Matthieu MM Damshcroder LJ Chinman MJ Smith JL Use of concept mapping to characterize relationships among implementation strategies and assess their feasibility and importance: results from the expert recommendations for implementing change (ERIC) study. Implement Sci. (2015) 10:109. 10.1186/s13012-015-0295-0 26249843 PMC4527340

[12] Emsley R. ChatGPT: these are not hallucinations—they're fabrications and falsifications. Schizophrenia (Heidelb). (2023) 9(1):52. 10.1038/s41537-023-00379-4 37598184 PMC10439949

[13] ChatGPT-3.5. Version 2023 (2023). (accessed June 27, 2023)

[14] Sandelowski M. Whatever happened to qualitative description? Res Nurs Health. (2000) 23(4):334–40. 10.1002/1098-240X(200008)23:4<334::AID-NUR9>3.0.CO;2-G 10940958

[15] Rybojad B Sieniawski D Rybojad P Samardakiewicz M Aftyka A. Pain evaluation in the paediatric emergency department: differences in ratings by patients, parents and nurses. Int J Environ Res Public Health. (2022) 19(4):2489. 10.3390/ijerph19042489 35206676 PMC8872586

[16] Brender TD. Medicine in the era of artificial intelligence. JAMA Intern Med. (2023) 183(6):507–8. 10.1001/jamainternmed.2023.1832 37115537

[17] Okorie CL Gatsby E Schroeck FR Ould Ismail AA Lynch KE. Using electronic health records to streamline provider recruitment for implementation science studies. PLoS One. (2022) 17(5):e0267915. 10.1371/journal.pone.0267915 35560153 PMC9106149

[18] Whalen M Maliszewski B Gardner H Smyth S. Audit and feedback: an evidence-based practice literature review of nursing report cards. Worldviews Evid Based Nurs. (2021) 18(3):170–9. 10.1111/wvn.12492 33512082

[19] Lyles CR Nelson EC Frampton S Dykes PC Cemballi AG Sarkar U. Using electronic health record portals to improve patient engagement: research priorities and best practices. Ann Intern Med. (2020) 172(11 Suppl):S123–9. 10.7326/M19-0876 32479176 PMC7800164

[20] Holmes JF Freilich J Taylor SL Buettner D. Electronic alerts for triage protocol compliance among emergency department triage nurses. Nurs Res. (2015) 64(3):226–30. 10.1097/NNR.0000000000000094 25932701 PMC4418031

[21] Obeid JS Beskow LM Rape M Gouripeddi R Black RA Cimino JJ A survey of practices for the use of electronic health records to support research recruitment. J Clin Transl Sci. (2017) 1(4):246–52. 10.1017/cts.2017.301 29657859 PMC5890320

[22] Watterson TL Ston JA Brown R Xion KZ Schiefelbein A Ramly E Cancelrx: a health IT tool to reduce medication discrepancies in the outpatient setting. J Am Med Inform Assoc. (2021) 28(7):1526–33. 10.1093/jamia/ocab038 33835183 PMC8661442

[23] Chary AN Brickhouse E Torres B Santangelo I Carpenter CR Liu SW Leveraging the electronic health record to implement emergency department delirium screening. Appl Clin Inform. (2023) 14(3):478–86. 10.1055/a-2073-3736 37054983 PMC10284630

[24] Da FC Pourhomayoun M Keeves D Lees AF Sarrafzadeh M Bell D Feasibility study of an EHR-integrated mobile shared decision making application. Int J Med Inform. (2019) 124:24–30. 10.1016/j.ijmedinf.2019.01.008 30784423

[25] Stanhope V Matthews EB. Delivering person-centered care with an electronic health record. BMC Med Inform Decis Mak. (2019) 19(1):168. 10.1186/s12911-019-0897-6 31438960 PMC6704707

[26] Jones LK Ladd IG Gregor C Evans MA Graham J Gionfriddo MR. Evaluating implementation outcomes (acceptability, adoption, and feasibility) of two initiatives to improve the medication prior authorization process. BMC Health Serv Res. (2021) 21(1):1259. 10.1186/s12913-021-07287-2 34801025 PMC8606066

[27] Kuske S Willmeroth T Schneider J Belibasakis S Roes M Borgmann SO Indicators for implementation outcome monitoring of reporting and learning systems in hospitals: an underestimated need for patient safety. BMJ Open Qual. (2022) 11(2):e001741. 10.1136/bmjoq-2021-001741 35437258 PMC9016397

[28] Willmeroth T Wesselborg B Kuske S. Implementation outcomes and indicators as a new challenge in health services research: a systematic scoping review. Inquiry. (2019) 56:46958019861257. 10.1177/0046958019861257 31347418 PMC6661793

[29] Anzelc M Burkhart CG Burkhart CN. Can artificial intelligence technology replace human scribes? Cutis. (2021) 108(6):310–1. 10.12788/cutis.0402 35167782

[30] Coiera E Kocaballi B Halamka J Laranjo L. The digital scribe. NPJ Digit Med. (2018) 1:58. 10.1038/s41746-018-0066-9 31304337 PMC6550194

[31] Falcetta FS de Almeida FK Lemos JCS Goldim JR da Costa CA. Automatic documentation of professional health interactions: a systematic review. Artif Intell Med. (2023) 137:102487. 10.1016/j.artmed.2023.102487 36868684

[32] Noorbakhsh-Sabet N Zand R Zhang Y Abedi V. Artificial intelligence transforms the future of health care. Am J Med. (2019) 132(7):795–801. 10.1016/j.amjmed.2019.01.017 30710543 PMC6669105

[33] Komorowski M Celi LA Badawi O Gordon AC Faisal AA. The artificial intelligence clinician learns optimal treatment strategies for sepsis in intensive care. Nat Med. (2018) 24(11):1716–20. 10.1038/s41591-018-0213-5 30349085

[34] Lam TYT Cheung MFK Munro YL Lim KM Shung D Sung JJY. Randomized controlled trials of artificial intelligence in clinical practice: systematic review. J Med Internet Res. (2022) 24(8):e37188. 10.2196/37188 35904087 PMC9459941

[35] Shen J Zhang CJP Jiang B Chen J Song J Liu Z Artificial intelligence versus clinicians in disease diagnosis: systematic review. JMIR Med Inform. (2019) 7(3):e10010. 10.2196/10010 31420959 PMC6716335

[36] Lim SM Shiau CWC Cheng LJ Lau Y. Chatbot-delivered psychotherapy for adults with depressive and anxiety symptoms: a systematic review and meta-regression. Behav Ther. (2022) 53(2):334–47. 10.1016/j.beth.2021.09.007 35227408

[37] Milnes-Ives M de Cock C Lim E Shehadeh MH de Pennington N Mole G The effectiveness of artificial intelligence conversational agents in health care: systematic review. J Med Internet Res. (2020) 22(10):e20346. 10.2196/20346 33090118 PMC7644372

[38] Birt L Scott S Cavers D Campbell C Walter F. Member checking. Qual Health Res. (2016) 26(13):1802–11. 10.1177/1049732316654870 27340178

