# Supplementary material for: A use case of ChatGPT: summary of an expert panel discussion on electronic health records and implementation science
Source: Front Digit Health. 2024 Oct 24;6:1426057. doi: 10.3389/fdgth.2024.1426057 (PMC11540825; doi:10.3389/fdgth.2024.1426057)
Supplement: Supplementary file 1 [file Supplementaryfile1.docx]

Generate a full scientific manuscript that includes a title, introduction, methods, results, discussion, and acknowledgements. The manuscript describes using ChatGPT in an expert panel session that addresses the electronic health record and implementation science. Do not include references.

Title: Include language that reflects incorporating ChatGPT to summarize and report an expert discussion on electronic health record and implementation science.

Introduction: Generate the introduction section that includes text that discusses the following topics: (1) a general discussion of how technology is transforming research and clinical practice; (2) opportunities of these technologies to support implementation science; (3) the EHR as a ubiquitous technology that could inform implementation science research; and (4) LLMs as a powerful tool for natural language summarization and generation. Include a final paragraph with the following text: “The objective of this manuscript is to present an expert panel session that incorporated ChatGPT to summarize and report on a session that addresses the electronic health record and implementation science.” Ensure the introduction section includes 3-5 paragraphs of text.

Methods: Write a methods section using language similar to other articles describing nominal group processes to describe the following material: This 1-hour session was part of a larger QUERI meeting that addressed implementation science topics. Fifteen implementation science experts attended our session to discuss the role of the EHR in supporting implementation science research. We presented Proctor's Implementation Outcomes framework as part of our background to the discussion, and we presented Implementations Strategies that were categorized by Waltz et al. We used three small break out groups to discuss two questions: (1) "How can the EHR support implementation strategies?"; and (2) "How can the EHR assess implementation outcomes?"

We had a dedicated participant scribe who summarized and recorded responses to these questions. While participants were discussing a third question (“What is the future of EHRs”), the scribe emailed the responses to the session lead (STR) who incorporated them as results into a detailed template (Appendix 1) that acted as a prompt for ChatGPT to write a full scientific manuscript (Appendix 2). Participants were not aware that their responses would be fed into ChatGPT until after the manuscript was generated. We used ChatGPT-3.5 architecture version May 25, 2023.

Once the manuscript was generated, it was distributed via email to the group along with an anonymous online survey that included Likert scale and free-text questions about processes and outcomes of the session, including perceptions of using ChatGPT to summarize and report on the discussion and the major themes that were generated.

We edited the manuscript to add survey findings, revise the text, and incorporate references (which had been intentionally omitted in the initial prompt. We include a marked-up version of the manuscript to demonstrate our changes (Appendix 3).

Results: Please summarize the following discussion:

Participants responded to the question "How can the EHR support implementation strategies?" with the following responses:

- Interactive assistance and feedback
- Stakeholder engagement using dashboards
- Generates data to support clinicians
- Engages consumers via patient portal (e.g., My HealtheVet)
- EHR as a way to change infrastructure (e.g., reminders, hard stops, etc.)
- Train the trainer
- Audit & Feedback – facilitation
- Case-finding dashboard and direct patient outreach
  - Finds patients, Sends letters
  - Reports, back-end data
- Audit & feedback – look at performance at local site and provide feedback to clinicians
- EHR as communication mode among teams (e.g., social work adding provider to the note)
- EHR to change workflows and learn from other providers
- Change infrastructure
- Support clinicians via decision support, (e.g., pharmacogenetics)
- The notion of tailoring to context – amazing how much the EHR is central to the notion of work.  Integral contextual piece in anything you’re implementing or facilitating (e.g., Cerner doesn’t map on to the VA’s access to care structure).
- EHR as a way to capture what you need to track the data for implementing what you need vs. EHR as a tool for 2-way communication with the patient and other stakeholders.
- Integrating of patient portal with the clinician interface. It’s not just a clinician and administrative tool.
- Train and educate stakeholder through simple messaging (but not through alerts)– example: clinical reminders.  Providers liked getting updating support
- Medication information that educates the provider (example)
- Keeping the autonomy of the providers
- In private sector there is no clinician to clinician discussion because it was not relevant to the coding / billing piece.

Participants responded to the question “How can the EHR assess implementation outcomes?” with the following responses:

- Depends on what you’re implementing – sometimes what you’re implementing is part of the EHR.
- Penetration uptake and uptake of intervention
- Can look at sustainability. If you can build in the outcomes into the structured data you can look at it after people have left.
- Can look at timeliness between referral to action or diagnostic testing to results
  - For treatments that need multiple visits (e.g., mental health) you can track if they are consistently coming to appts.
- Do we have opportunity to tailor EHR to capture some of these outcomes?
  - Many are text & not structured so hard to capture - changes in well-being
  - Can research people at table with EHR get some of these things added
    - If so – who will be inputting this data? Will providers actually be able to code/provide this data or who will enter?
- Data on acceptability
- Costs tracking, trends
- Timeliness
- Use patterns within the EHR – how often specific parts engaged – feasibility, penetration/reach and adoption
- Sustainability – window into, accessing specific things in record
- Acceptability - reach beyond champions?
- Monthly monitor – trends in use, identifying plateaus (sustainability)
- Characteristics of patients, providers, clinics
- Data on acceptability
- You collect all the data!!! You can see and quickly see trends and outcomes
- Anything that relies on the notes, however, is a lot harder.  This is an example of what the EHR is not good at helping you with.
- You can observe work in how people use the EHR itself.  Lights on lights off.  Workload can be measures.    It’s a space in which people are working and you can look at workflow from that perspective.  Will it have any impact in the way people interface with the EHR.
- Pain in the EHR — for years we needed to collect pain scores, we wanted them to be low.  This is part of how the opioid crisis happened. It changed care dramatically.  Once we collected the data we decided we wanted to do things.  It CHANGED care.  We’ve now dropped a lot of pain score data.  “COVID solved pain in the VA”.  Pain doesn’t belong to a single specialty. So who’s doing it?  How are nurses charting it? Are different specialties capturing it differently? By looking at what’s getting captured in the EHR, it helps you think about
- EHR to some extent drives the prioritization of what data gets collected (and as a follow0up , what gets captured gets attended to.

Participants responded to the question “How will future EHRs further support implementation science?” with the following responses:

- How much flexibility to change the structure to support implementation efforts?
- What can we do if we could change EHR: Use AI to make the clinician’s life easier. Improve implementation of shared decision making. Can EHR take ease the burden of this data entry and/or assessment?
- U Penn work – using EHR to prevent clinicians from certain prescribing or treatments – system makes it harder for them to prescribe certain meds by making them having to go through further steps (nudges to do things in a certain way).
  - Create barriers to prescribing in targeted ways – prompts and reminders based on what clinically is best practice
  - Targeted decision making
  - Reducing provider burden to improve focused care/specific care.
- AI, giving providers more real-time information, choices
- Assessing the impact entails unpacking the black box of AI
- Problems with healthcare system and society misinformation
- How could EHR be a broker of quality information? Ability of EHR to share information with patients – support patient engagement – transparency about what’s in the EHR, shared data visualizations to engage patients. Pull EHR info to tailor auto chatbot supports
- EHR as input to population health – ability to pull broad outcomes, characteristics, data
- Visualization of data to inform policy
- Could EHR become part of the care team? Could it supplant/replace the care team?
- Future implementation science could be more about data management and analysis versus people management.
- Will support more integration of patient goals and priorities in all of care planning
- Integration of ChatGPT to minimize workload for provider.  Generate text that has high emotional burden to compile, (e.g., give me the language of what to say to someone who doesn’t believe in vaccines”)
- Increasingly data will be put in from sources beyond the clinician (wearables, remote weight scales for example)
- Recurring theme:  what can be done without having to talk to another human — we need to move into the other direction!
- Can help redesign the workflow (who does what part of clinical care — does X have to be handled by primary care, for example?  Offloading things out of the care visit so the visit can be more conversational instead of box checking)
- How can the EHR help flip the switch in patient mentality of “my doctor takes care of me” and all this other stuff is in the way of me talking to my provider

Discussion: Write a discussion section that includes text the following content: (1) note the importance of a discussion on EHRs and implementation science and the novelty of using ChatGPT; (2) provide a high level summary of findings; (3) include 1-2 paragraphs on general themes that transcended responses to individual question and reflect the relevance of EHRs to implementation science; (4) write 1-2 paragraphs on using LLMs to summarize an expert panel session, including the positive elements of rapidly digesting and presenting data, the potential negative elements of not using human methodologists to analyze and interpret the findings, and the acknowledgement that this was a fairly simple use case with straightforward and structured data; (5) include a limitations section; (6) include a concluding paragraph that summarizes the manuscript and offers implications for future implementation research. Ensure the discussion section includes 6-8 paragraphs.

Acknowledgement: Include the VA disclaimer. State that this project was funded by the VA QUERI Evidence, Policy, and Implementation Center (VA QUERI EBP 22-104).
